# Opportunities and Limitations of Molecular Methods for Studying Bat-Associated Pathogens

**DOI:** 10.3390/microorganisms10091875

**Published:** 2022-09-19

**Authors:** Silvia Zemanová, Ľuboš Korytár, Jana Tomčová, Marián Prokeš, Monika Drážovská, Łukasz Myczko, Piotr Tryjanowski, Gréta Nusová, Alicja Matysiak, Anna Ondrejková

**Affiliations:** 1 Department of Epizootiology, Parasitology and Protection of One Health, University of Veterinary Medicine and Pharmacy in Košice, 041 81 Košice, Slovakia; 2 Department of Zoology, Poznań University of Life Sciences, 60-625 Poznań, Poland; 3 Institute of Biology and Ecology, Faculty of Science, Pavol Jozef Šafárik University in Košice, 041 54 Košice, Slovakia

**Keywords:** bats, PCR, viruses, diseases, sequencing

## Abstract

Bats have been identified as reservoirs of zoonotic and potentially zoonotic pathogens. Significant progress was made in the field of molecular biology with regard to infectious diseases, especially those that infect more than one species. Molecular methods, sequencing and bioinformatics have recently become irreplaceable tools in emerging infectious diseases research and even outbreak prediction. Modern methods in the molecular biology field have shed more light on the unique relationship between bats and viruses. Here we provide readers with a concise summary of the potential and limitations of molecular methods for studying the ecology of bats and bat-related pathogens and microorganisms.

## 1. Introduction

Bats (order Chiroptera) are a group of mammals with unique anatomy and physiology, which predispose them to fulfill a variety of ecological functions [1]. Known as the only mammals capable of sustained flight [1], bats occur ubiquitously except in Antarctica [2].

The impact of bats as reservoirs of zoonotic viruses has been defined in numerous studies [3,4,5,6]. Infectious pathogens, especially those that infect more than one species, are a complex subject that has recently captured the attention of many researchers. This impact is underlined by the fact that about 75% of zoonoses are spread to humans from wildlife [7,8,9,10,11,12,13]. One of the most important bat-borne zoonoses is bat rabies. Human–bat interactions are now the source of the majority of locally acquired human lyssavirus infections in many high-income countries without hematophagous or ‘vampire’ bat species [14]. On the other hand, experimental European bat lyssavirus (EBLV) infections in other mammals have revealed only low susceptibility of foxes by the intramuscular route [15]; an experimental EBLV infection of sheep resulted only in a peripheral abortive infection without neurological signs [16]. As for known or supposed natural infections, evidence suggests that Hendra virus and Nipah virus may have been transmitted from bats to other mammals beside humans [17,18,19]. Hendra virus was not highly contagious in experimental conditions [20].

Besides their role in emerging infectious diseases (EID), bats also fulfil indispensable roles in the functioning of the ecosystem [1,21]. While nectarivorous species are important pollinators of fruit-bearing trees and frugivorous bats play a role in the process of reforestation [22,23,24], insectivorous species are significant predators of insects [1,21,25]. Whenever bat populations experience a higher mortality rate, the decline in numbers may affect the whole ecosystem, even economics [26]. The guano of bats is rich in nitrogen and is used as fertilizer [27]. In several countries, bats are listed as species protected by local laws. Therefore, research of bat-related EID is ruled by ethical committees; specific bat biological sample availability is limited in some cases, as non-invasive sampling methods must often be preferred in these species [28].

**Figure 1:**
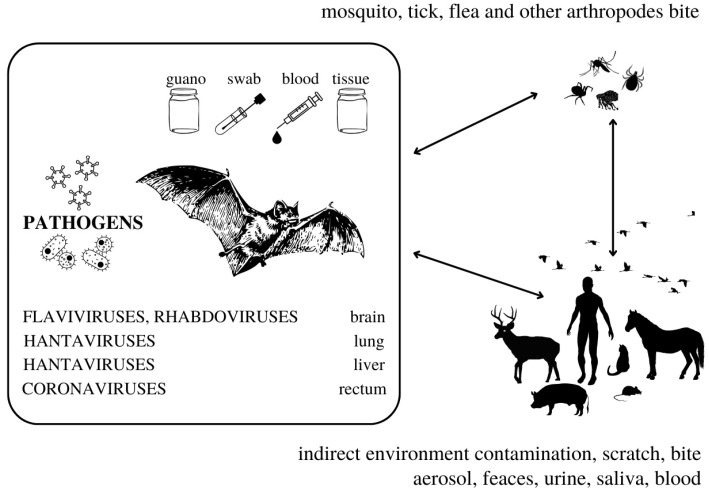
The complexity of bat-borne pathogens transmission. The information in this figure was compiled using studies cited in this review.

A definitive reservoir species for a pathogen, according to Mlera et al. [29], may be defined in two ways: a species from which it is possible to isolate infectious pathogens, or a species that demonstrates high positivity for the said pathogen in surveillance studies [29]. Potential reservoir species may be proven to be seropositive and/or viral RNA may be present in their tissues [29]. On the other hand, Villarreal [30] states that the term persistence (including latent and chronic infection) better describes some instances of “symbiotic virus–host relationships” that are different from a simple reservoir of viruses. Since the invention of the first molecular methods, a reservoir status has been described in bats from which pathogens may be transmitted by many different routes (Figure 1) [4,6]; some viruses seem to persist in a few bat species as well; cases of possible viral elements inserted in bat genome have been identified [31,32]. Reservoir species are responsible for maintaining a pathogen in a region in the long term and for transmitting it to other species of concern [33]. To elucidate complex patterns of infection, long-term serological data with advanced analytical tools have proven necessary [34].

Bats have been identified as an important reservoir species for many viral and a few bacterial pathogens [27]. Numerous studies have linked bats as potential reservoir species of lyssaviruses, henipaviruses, hantaviruses and coronaviruses [2,12,35,36,37,38,39,40,41,42,43,44,45,46,47]. The authors Cadar et al. [48], Pilipski et al. [49] and Kading et al. [50] report flavivirus detection in bats. Other viruses, such as adenoviruses, circoviruses, picornaviruses, papillomaviruses, herpesviruses, etc., were also detected in bats [12,51,52]; however, the role of bats as reservoirs in these cases still remains to be elucidated.

Since these EIDs have been linked to bats, and the most recent SARS-CoV-2 pandemic seems to also involve bat SARS-related coronaviruses [47,53,54]; therefore, the bats serve as a focal point of our mini-review. We address qualitative rather than quantitative aspects of the research subject. Our mini-review highlights the works that demonstrate the potential, as well as limitations, of molecular methods for studying the ecology of bats and bat-related microorganisms.

## 2. Molecular Methods in Bat EID Research

Molecular methods, sequencing and bioinformatics have recently become irreplaceable tools in EID research and even outbreak prediction. As the number of available studies implies, bats seem to be linked more to research concerning RNA viruses than DNA viruses [55,56]. Although several DNA viruses were detected in bats [12,57,58], these do not seem to have a significant impact on bat populations; neither do these viruses seem to be the etiological agent of a zoonotic disease outbreak.

RNA isolation, transcription and a variation of PCR [59] are therefore used quite often as first-choice methods in virus detection in bats, and sequences of the most common viruses are available in databases like GenBank.

### 2.1. PCR Leading to Discoveries

A standard PCR protocol followed by sequencing of purified amplicon is a versatile way of both identifying well-known viruses and finding new viruses. Examples of the relevance of this approach are studies similar to Straková et al. [44], who identified a novel hantavirus in bat liver tissue, performing a total RNA extraction (QIAamp viral RNA Mini Kit or Qiazol/triazol method) from internal organ tissues collected from dead individuals and proceeding to screen tissue samples for hantavirus RNA presence and sequencing of PCR amplicons (from a broad-spectrum RT-PCR). To determine the entire genomic sequence of this novel virus, IonTorrent HTS analysis was performed.

Cadar et al. [48] identified Usutu virus RNA in bat brain tissue by performing both total DNA and RNA extraction from tissues, then subjecting extracted nucleic acids to RT-PCR. Amplicons were sequenced directly and these sequences were used for phylogenetic analysis. Similar to Straková et al. [44], a complete genome sequence of the bat Usutu virus was then obtained from bat brain tissue using a set of primers designed from multiple comparisons of Usutu virus genomes available in databases [48].

A novel bat-borne hantavirus was detected in samples of bat lung tissue by Arai et al. [60], using a heminested L-segment primer set and a nested S-segment primer set. Their results might be indicative of a host-switching event [60], which might help understand the ecology of these viruses.

Luo et al. [61] collected 1044 bat brains and 3532 saliva swab samples to search for molecular evidence of rhabdoviruses. For initial screening, they used a previously described combined real-time reverse transcription PCR (RT-qPCR) that includes a probe-based RT-qPCR for pan-rabies virus detection and another pan-lyssavirus RT-qPCR [61]. Their effort led to the discovery of six new rhabdoviruses, the sequences of which were determined by next-generation sequencing and confirmed by Sanger sequencing. One of the tentative rhabdovirus species identified in this study clustered with two insect-related viruses; the authors did not exclude a possible role for arthropods in the life cycle of the identified bat viruses [61]. Although these rhabdoviruses were considered unlikely to present a high risk of spillover events, further information about transmission and shedding of these viruses in bats is needed to determine their zoonotic potential [61].

A wide range of rhabdoviruses was discovered in bats and bat parasites by Aznar-Lopez et al. [62], who performed a nested RT-PCR on 1488 oropharyngeal swabs from bats.

### 2.2. Coronaviruses Are Found Abundantly Using Non-Invasive Methods

Bat guano was successfully used as a sample for coronavirus detection in a long-term study performed by Lo et al. [63]. The authors obtained 512 fecal samples over the course of 4 years. RNA was isolated from these samples and carried out a nested PCR for coronavirus detection. Analysis of the sequences obtained in their study revealed that the detected coronaviruses belonged to the genera *Alphacoronavirus* and *Betacoronavirus*, some of which were grouped with the SARS-like coronavirus clade [63]. Using non-invasive sampling methods, such as guano collection, can lead to significant discoveries in bat-borne virus research; identification of non-invasive or less invasive samples such as saliva, urine or feces is also a key element in terms of bat conservation [61].

Anthony et al. [64] screened 606 bats for coronaviruses, collecting blood, oral and rectal swabs. Their method of choice was PCR using broadly reactive consensus primers.

### 2.3. Modern Sequencing Methods and Viral Diversity

Sanger’s sequencing method [65] and its more modern modifications belong to the first generation of sequencing methods. This method has been reported to still be the most accurate. It is widely accepted that NGS variants need to be validated with the gold standard Sanger sequencing technique prior to reporting, even though both the costs and turnaround time of this approach are considerable [66]. Another question needing an answer is how much can be concluded about the reservoir status of the bat even after successfully confirming the presence of a pathogen nucleic acid fragment in the samples. To fully confirm the reservoir status of a species, much more data is required; however, depending on the location of the detected pathogen fragment in the body of the host, the direction of further research into the virus–host relationship can be determined.

It was pointed out that traditional Sanger sequencing can only be applied to individual samples (or a low number thereof), which makes the method too painstaking for processing complex samples, especially for large-scale studies [67].

In the literature, the term next-generation sequencing (NGS) is often used to describe sequencing platforms other than those based on the Sanger method (pyrosequencing, sequencing by synthesis, ligation and two-base coding) [68]. NGS, also known as massively parallel or deep sequencing, is characterized by the ability to sequence millions of short DNA fragments in parallel [69]. NGS has proved to be a very efficient method to determine the virome of mammals, including bats, such as the extensive and highly efficient study performed by Wu et al. [12] in which a broad range of viruses, most of them novel, were identified in swab samples from 4440 bats. Metagenomics, defined as the direct genetic analysis of genomes contained with an environmental sample [70], has led to important findings, some of which encompass novel and/or potentially zoonotic viruses in bats [71,72,73,74,75]. Library preparation, being an important part of metagenomics, has also been pivotal in the research of full-genome sequencing of novel bat-associated viruses [51,52]. Wu et al. [12] used a series of sequence-independent RT-PCR, sequence-based PCR and specific nested PCR amplification methods, along with viral library construction and NGS, to analyze the viral community in the sampled bat species. Wu et al. [12] stated that the purpose of their study was to survey the ecological and biological diversities of viruses residing in these bat species, to investigate the presence of potential bat-borne zoonotic viruses and to evaluate the impacts of these viruses on public health. Recently, metagenomics has found a potential use in diagnostics [76] and surveillance [77].

Broad-spectrum studies, like Wu et al. from 2016 and 2018 [12,78], provide molecular proof for viral diversity in mammals. As the authors Wu et al. [78] conclude, combined with previous bat virome data, such studies greatly increase our knowledge of the viral community in wildlife in a densely populated country in an EID hotspot and continued efforts in viral discovery in these and other mammalian hosts may reveal greater diversity of viral lineages.

### 2.4. In Silico Analyses May Reveal Bases for Further Research

While it has long been known that the eukaryotic genome contains endogenous retroviruses, it was surprising to discover that sequences of RNA viruses that do not make a DNA intermediate and do not usually enter the nucleus are also present in eukaryotic genomes [79,80]. A useful collective term reflecting their fragmentary nature, EVE (endogenous viral elements), has been coined by Katzourakis and Gifford [24]; Holmes [80] uses this term to refer to all endogenous viruses regardless of taxonomy.

Using an initial PCR screening and phylogenetic analyses, Horie et al. [81] demonstrated that bats of the genus *Eptesicus* carry an inheritable endogenous bornavirus-like L (EBLL) element in their genome. Representatives of the genus *Eptesicus* occur in a wide geographical area including the northern hemisphere within Europe, Asia and the Americas [82]. These findings provide novel insights into the co-evolution of RNA viruses and mammalian species [81].

Taylor et al. [32] performed first an in silico screening of NIRV (non-retroviral integrated RNA viruses) in bat sequences; they further tested the presence of integrated copies of DNA-based filovirus sequences in the two species with the highest copy number, the wallaby species *Macropus eugenii* and the bat species *Myotis lucifugus*. They designed PCR primers from the mammalian genomic sequence belonging to the longer identical sequences and performed amplification of these segments in individuals of these species other than those used in existing genomic projects. The sequence from *Macropus eugenii* had only one mutation compared to the sequence from the mentioned genome project [32]. Taylor et al. [32] subsequently tested samples of bats of *Myotis lucifugus* and *Eptesicus fuscus* for the presence of these sequences. In all cases, the similarity of the new sequences was consistent with the hypothesis of integration of a filovirus-like copy of DNA into mammalian genomes. This laboratory finding supported their hypothesis expressed after an in silico examination of the genomic database; the phylogenetic analysis and sequencing performed in this work is consistent with the hypothesis of integration of filoviral elements into mammalian genomes [32]. Phylogenetic evidence suggests that the direction of transfer was from viral to mammalian genome [32].

Integrated viral elements have been found not only in bats but also in the genome of arthropod vectors [31,32]. Katzourakis and Gifford [31] reported that when these nucleotide sequences are fixed in the genome of a mammalian species, they can provide phylogenetic benefits to the species. These authors are of the opinion that the low frequency of recording events of the integration of the viral genome into the host genome is due to the low number of sequenced genomes of species and individuals and not to the fact that they are not retroviruses or viruses replicating in the nucleus of cells. Integrated viral elements can cause the host immune system to recognize these viral transcripts as “self” if they co-evolve with a mammalian host [31,83].

A more recent study by Skirmuntt et al. [84], which focuses heavily on bat immunology as a factor influencing EIDs, corroborates the conclusions of the aforementioned authors; the in silico method seems to continue gaining importance within the molecular biology field.

## 3. Discussion

To determine a working solution to the threat posed by EID, it seems to be important to consider what a reservoir is and also to consider the evolutionary history of reservoir species. Several authors have compiled different EID management frameworks over the course of the past years [85,86,87]. It is necessary to include bat immunology [84], biological, social and environmental science and even mathematics [86] in a ‘one health’, multidisciplinary approach [85] to EID research. Deeply understudied social and environmental questions should also be taken into account when dealing with EIDs [86]. Holmes [80] describes in some cases the relationships between a macro-organism and a virus as an intense “arms race”. The author concludes his work by stating that the discovery of EVE has raised some questions, including whether these can be beneficial to the host cells. Thorough, systematic, long-term studies comparing bat viromes to the viromes of other reservoir species, including research in EVEs and phylogenetic studies, could provide an insight into the true “molecular” nature of their reservoir/viral persistence status. Modern metagenomics and high-throughput sequencing methods, combined with PCR screening and phylogenetic analysis, have proven their use in providing an insight into the EIDsʼ true identity by identifying their molecular nature. These methods could, when combined, shed light on the relationships between viral hosts, reservoirs and viruses—in other words, to the ecology of viruses. Some zoonotic viruses are part of the ecosystem within the phenomenon of natural outbreaks; they are well adapted to reservoir macro-organisms. Since the most effective place to address such zoonotic threats is at the wildlife–human interface [86], public health infrastructure will also remain one of the most important factors involved in prevention and control of EIDs, regardless of future progress in laboratory diagnostics.

Studying bat ecology and evolution will also provide a better understanding of zoonotic virus–host dynamics, as well as the long-term co-evolutionary process between hosts and some viruses [84]. Viruses and parasites in general are a part of the ecosystem, especially those that display the phenomenon of natural outbreaks. While straightforward pathogen detection methods (sample collection, PCR, sequencing and phylogenetic analysis) are indispensable; bioinformatics hand-in-hand with ecology are necessary to analyze the “bigger picture”. Even hyperparasitism has been described in bats and their parasites, suggesting a potential for new discoveries in this field [88].

PCR and sequencing belong to the most relevant techniques in identifying EID outbreaks. In silico methods, as demonstrated by cited authors, have recently become equally relevant in EID management, as they can shed more light onto the underlying reasons behind the origin of zoonotic diseases. Input is needed from both human and veterinary medicine, along with deeper immunological, microbiological and bioinformatic insights.

Metagenomic analyses of non-invasive samples can provide information useful for surveillance [71,76] and early detection of viral zoonoses [71]. However, the nature of the sample affects its informative value. The mere fact that the nucleic acid of a virus is detected in, for instance, bat feces may indicate bat involvement in the ecology of the virus and may therefore point the direction for further research but will not inform exhaustively about their reservoir status. This is supported by the fact that various insect virus nucleic acids were detected in insectivorous bat guano [74]. Insectivorous bats have been proven to have a far more abundant virome than frugivorous bats [12]. In their study, Vicente-Santos et al. [89] conclude that bats serve only as dead-end hosts for Dengue virus, the presence of this virus being the result of ingestion of a positive mosquito vector. Several other studies suggested that bat diet and ectoparasite load may be related to the detection of arthropode viruses in samples from bats. A broad range of “dietary viruses” [57,90] have been discovered in bat guano [57], which are the result of passive transport of insect virome, including mosquito virome, through the gastrointestinal tract, while until now it remains unclear if these viruses can cross the intestinal wall and infect bats [90]. This arthropod–virus–bat relationship has been described both in frugivorous [90] and insectivorous bats [57]. Vicente-Santos et al. [89] combined PCR and serology from both bats and mosquitoes to reach their conclusion.

Worldwide, the access to different sample types is unbalanced, mostly because bats are protected by law in most European countries. To ensure proper bat conservation, non-invasive samples should be preferred. In future, bat-related EID research will benefit from finding ways to enhance the informative value of non-invasive samples, for example a systematic approach to metagenomics analysis of multiple non-invasive sample types collected from the same individual, combined with the research of viromes of bat-related arthropods and their immediate environment (Table 1). Collecting samples from the environment and other mammals may provide further information about bats as reservoirs [91]. 

## Figures and Tables

**Table 1:** Summary of use of the molecular methods in research of bats. The table was composed using the studies cited in this review.

Method Used	Sample Size	Information Obtained	Advantages	Disadvantages
PCR + Sanger sequencing from tissues	Smaller	Ecology of the pathogen; bat infected	Lower initial costs	Less chance of finding new viruses
PCR + Sanger sequencing from swabs/guano	Smaller	Ecology of the pathogen; possible spread of the pathogen from bats	Lower initial costs	Less chance of finding new viruses
Metagenomic analysis (tissues)	Larger	Virome, infection	High informative value, infection	Price
Metagenomic analysis (swabs/guano)	Larger	Virome	High informative value, ecology	Price

## Data Availability

Not applicable.
